# Functional Analysis of PsAvr3c Effector Family From *Phytophthora* Provides Probes to Dissect SKRP Mediated Plant Susceptibility

**DOI:** 10.3389/fpls.2018.01105

**Published:** 2018-07-25

**Authors:** Ying Zhang, Jie Huang, Sylvans O. Ochola, Suomeng Dong

**Affiliations:** ^1^Department of Plant Pathology, Nanjing Agricultural University, Nanjing, China; ^2^Key Laboratory of Integrated Management of Crop Diseases and Pests, Ministry of Education, Nanjing Agricultural University, Nanjing, Chxsina

**Keywords:** PsAvr3c, *Phytophthora*, effector family, virulence, SKRP, alternative splicing

## Abstract

PsAvr3c is an effector identified from oomycete plant pathogen *Phytophthora sojae* that causes soybean root and stem rot disease. Earlier studies have demonstrated that PsAvr3c binds to a novel soybean spliceosomal complex protein, GmSKRP, to reprogram the splicing of hundreds of pre-mRNAs and consequently subvert host immunity. PsAvr3c family genes are present in some other *Phytophthora* species, but their function remains unknown. Here, we characterized the functions of *PsAvh27b* (*PsAvr3c* paralog from *P. sojae*), *ProbiAvh89* and *PparvAvh214* (orthologs from *P. cinnamomi* var. *robiniae* and *Phytophthora parvispora*, respectively). The study reveals that both PsAvh27b and ProbiAvh89 interact with GmSKRPs *in vitro*, and stabilize GmSKRP1 *in vivo*. However, PparvAvh214 cannot interact with GmSKRPs proteins. The qRT-PCR result illustrates that the alternative splicing of pre-mRNAs of several soybean defense-related genes are altered in *PsAvh27b* and *ProbiAvh89* when over-expressed on soybean hairy roots. Moreover, PsAvr3c family members display differences in promoting *Phytophthora* infection in a SKRP-dependent manner. Overall, this study highlights that the effector-mediated host pre-mRNA alternative splicing occurs in other pathosystems, thus providing new probes to further dissect SKRP-mediated plant susceptibility.

## Introduction

*Phytophthora*, a genus of plant pathogen oomycetes, cause many destructive crop diseases and result in considerable losses in agriculture and economy (Kroon et al., 2012). These pathogens are notoriously difficult to manage due to their capability to evolve fast to escape field resistance (Tyler, 2007). A well-characterized species is *Phytophthora* *infestans*, the causal agent of late blight disease, a major global threat to potato (*Solanum tuberosum*) and tomato (*Solanum lycopersicum*) production (Fry et al., 2015; Kamoun et al., 2015). *Phytophthora sojae*, another model research pathogen, induces soybean root and stem rot, resulting in significant economic losses every year around the world (Tyler, 2001). Understanding the pathogenesis of these pathogens is critical to developing durable plant resistance.

One of the important virulence mechanisms of *Phytophthora* species is their ability to deliver a range of secreted proteins (effector) into the host cells to subvert plant immunity. The best studied *Phytophthora* effector proteins belong to a so-called RxLR effector family. The RxLR effectors have a signal peptide followed by a N-terminal conserved RxLR (Arg-any residue-Leu-Arg) motif (Morgan and Kamoun, 2007). Many studies have demonstrated that RxLR effectors play roles in suppressing plant immunity through distinct ways. One way is to block host gene expression machinery. For example, *P. sojae* effector PsAvh23 enhances plant susceptibility through reducing the levels of GCN5-mediated H3K9 acetylation to suppress plant defense gene expression (Kong et al., 2017). Signaling transduction suppression is another common strategy, *P. infestans* effector PexRD2 suppress plant immunity through disruption of the MAPKKKε dependent signaling pathways (King et al., 2014). Moreover, effector could act in a plant hormone manipulation manner. For instance, *P. infestans* effector PexRD24 enhances plant susceptibility by attenuating jasmonic acid and salicylic acid-mediated transcriptional responses of the host plant (Boevink et al., 2016). These results demonstrate efficient but diversed virulence functions of *Phytophthora* RxLR effectors.

Previously, we identified *P. sojae* effector PsAvr3c that could be recognized by soybean cultivars that carry the cognate resistant gene *Rps3c* (Dong et al., 2009). Genome investigation indicated that besides the two copies of *PsAvr3c*, another *PsAvr3c* homologous gene, *PsAvh27b*, is also present in the vicinity of *PsAvr3c* loci. However, PsAvr3c rather than PsAvh27b triggers cell death on plants carrying *Rps3c* (Dong et al., 2009). Moreover, knockout of PsAvr3c in *P. sojae* strain P6497 results in a gain of virulence toward Rps3c soybean. However, these knockout mutants exhibited reduced virulence on susceptible soybeans, suggesting that PsAvr3c is required for full virulence of *P. sojae*. In addition, PsAvr3c carries a functional nuclear localization signal (NLS) peptide which accumulates PsAvr3c in the nucleus thus enhancing host susceptibility. We previously demonstrated that PsAvr3c binds to and stabilize soybean serine, lysine and arginine-rich protein (GmSKRPs), a novel plant spliceosome component that is involved in the alternative splicing of plant pre-mRNA (Huang et al., 2017). GmSKRPs is a negative regulator of plant immunity, and ectopic expression of GFP-GmSKRP1 in *N. benthamiana* also promoted *P. capsici* colonization, PsAvr3c and GmSKRP1 work through same genetic pathway (Huang et al., 2017). Interestingly, more than four hundred genes are differentially spliced in both GmSKRPs and PsAvr3c expressing lines, including soybean resistance related genes such as NAC and WRKY transcription factors (Huang et al., 2017). Intriguingly, *PsAvr3c* homologous genes are also present in other *Phytophthora* species. However, whether PsAvr3c family members from other *Phytophthora* species share similar virulence function is entirely unknown.

In this study, the functional analysis of *PsAvr3c* homologs from several other *Phytophthora* species revealed the conservation of effector proteins that act in PsAvr3c-similar manner. This study suggests that effector-mediated host pre-mRNA splicing change may act as a conserved virulence function in a range of *Phytophthora* species. Furthermore, the study also highlights the functional diversity of PsAvr3c thus providing us with valuable tool to further dissect the mechanisms of SKRP-mediated pre-mRNA alternative splicing in plant immunity.

## Materials and Methods

### Plant and Microbe Cultivation

*Nicotiana benthamiana* plants were grown in a greenhouse for 5–6 weeks under a 16 h day at 25°C and 8 h night at 22°C. Etiolated soybean seedlings were grown at 25°C without light for 5–6 days before inoculation. *P. sojae* (P6497) and *P. capsici* (Pc35) strains were routinely maintained on 10% vegetable (V8) juice medium at 25°C in the dark.

### GenBank Accession Numbers

*ProbiAvh89* (MH450044); *P. pistAvh226* (MH450043); *P. parvAvh214* (MH450045); *P. niedAvh208* (MH450046); *P. cijaAvh190* (MH450047); *P. vignAvh281* (MH450048). We harvested the full sequences of PsAvr3c and PsAvh27b from the publication paper (Dong et al., 2009).

### Plasmid Construction

The *PsAvh27b* gene was cloned using cDNA from *P. sojae*, and *ProbiAvh89*, *PparvAvh214* genes were artificially synthesized through the given amino acid sequence (Supplementary Table 1). All of these genes without a signal peptide were amplified using a combination of primers (Supplementary Table 2), and then ligated into pBINGFP2 (a plasmid containing green fluorescent protein (GFP) with the In-Fusion HD Cloning Kit (Clontech, Mountain View, CA, United States). The resulting recombinant plasmids were transformed into *Agrobacterium rhizogenes* K599 or *A. tumefaciens* GV3101 using the freeze-thaw method. The validated transformants were then used for transient expression of the corresponding effector genes into *N. benthamiana* or soybean using previously described protocols (Kereszt et al., 2007). *PsAvr3c* and *PsAvh27b* (without signal peptide and RXLR-dEER motif), and, *ProbiAvh89* and *PparvAvh214* (without signal peptide) genes were amplified using the combination of primers (Supplementary Table 2), and ligated into the pET32a vector (containing His tag) for pull-down assays, GmSKRPs was inserted into the pGEX-4T-2 vector (containing GST tag) (GE Healthcare Life Science). Individual colonies for each construct were tested for inserts by PCR, and selected clones verified via sequencing.

### *In Vitro* GST Pull-Down Assays

pET32a empty vector, His-PsAvr3c, His-PsAvh27b, His-ProbiAvh89, His-PparvAvh214, GST empty vector and GST-GmSKRPs were expressed in *E. coli* strain Rosetta2 respectively. The pull-down assay was performed using ProFound pull-down GST protein-protein interaction kit (Pierce) according to the manufacturer’s instructions. The soluble total GST-fusion proteins were incubated with 25 μL glutathione-agarose beads (Invitrogen) for 5 h at 4°C. The beads were washed three times and then incubated with 1 mL of bacterial lysates containing His proteins for another 4 h at 4°C. The beads were then rewashed three times, and the presence of His proteins was detected by western blot using anti-His antibody.

### Transformation of Soybean

Soybean cotyledons were inoculated with *A. rhizogenes* K599 carrying pBINGFP2, pBINGFP2-PsAvr3c, pBINGFP2-PsAvh27b, pBINGFP2-ProbiAvh89, and pBINGFP2-PparvAvh214. Individual cotyledons were collected from 6-day-old soybean seedlings. Detached cotyledons were surface-sterilized with 70% ethanol before a small, roughly circular (diameter = ∼0.4 cm) cut was made in each cotyledon ∼0.3 cm from the petiole end. The wounded cotyledons were then transferred to a sterile Petri plate containing 0.8% agar. *A. rhizogenes* cells grown in LB medium supplemented with kanamycin were washed and resuspended in 10 mM MgCl_2_ to a final concentration of OD_600_ = 0.6. Twenty microliters (20 μL) of the cell suspension was directly inoculated onto the wound site of each cotyledon. The inoculated cotyledons were incubated in a growth chamber at 25°C under high humidity with a 16h/8h light/dark regime. GFP-tagged PsAvh27b, ProbiAvh89 and PparvAvh214 or GFP were expressed under the control of the CaMV 35S promoter. Fluorescence microscopy was used to select GFP, PsAvh27b, ProbiAvh89, and PparvAvh214 expressing roots. Green fluorescence was detected in hairy roots using a fluorescence stereomicroscope (Leica MZ FLIII, Wetzlar, Germany) with a GFP2 filter (excitation 480/40 nm, emission 510 nm). The expression of PsAvh27b, ProbiAvh89, and PparvAvh214 proteins in hair roots was confirmed by western blotting.

### qRT-PCR Analyses and Measurement of Splicing Efficiency Ratio

The RNA was quantified using a spectrophotometer (ND-1000; NanoDrop, Wilmington, DE, United States). Spectrophotometric analysis was used to determine the yield and purity of total RNA and make sure the ratio of OD260/280 is between 1.9 and 2.0. Agarose gel electrophoresis was used to test whether there existed RNA degeneration. To eliminate contaminating genomic DNA in the RNA samples, 3 μg of total RNA was treated with two units of RNase-free DNase I (Takara Bio Inc., Otsu, Japan) at 37°C for 30 min. First-strand cDNA was synthesized using Moloney murine leukemia virus reverse transcriptase (RNase-free) and an oligo(dT) 18 primer (Invitrogen, Carlsbad, CA, United States). qPCR was performed in 20 μl reactions containing 20 ng of cDNA, 0.2 mM gene-specific primer or the reference actin gene, 10 μl of SYBR Premix ExTaq (TaKaRa) and 6.8 μl of deionized water. PCR was performed on an ABI Prism 7500 Fast Real-Time PCR System (Applied Biosystems Inc., Foster City, CA, United States) under the following conditions: 95°C for 30 s and 40 cycles at 95°C for 5 s and 60°C for 34 s, followed by a dissociation step, that is, 95°C for 15 s, 60°C for 1 min and 95°C for 15 s.

The splicing efficiency ratio was calculated by determining the level of spliced RNA normalized to the level of unspliced RNA. The spliced primers were designed to measure the intron-spliced isoform expression, that crossed exon–exon junctions, while the unspliced primers were designed to span the intron–exon junction in order to measure the intron-retained isoform expression. Data were shown as average fold-change over the splicing efficiency ratio from three biological repeats.

### Plant Inoculation

For assays of *P. capsici* infection on *N. benthamiana* leaves, co-expression of GFP empty vector, GFP-PsAvr3c, GFP-PsAvr3C^M4^, GFP-PsAvh27b, GFP-ProbiAvh89, and GFP-PparvAvh214 with GFP-GmSKRP1 proteins were confirmed by western blotting using an anti-GFP antibody, with pBinGFP2 empty vector and GFP-PsAvr3C^M4^ as the negative control and GFP-PsAvr3c as the positive control, respectively. *P. capsici* isolate LT263 was used for infection of *N. benthamiana* leaves. The same size mycelial plugs were obtained using a 6 mm cork borer and used to inoculate the abaxial surface of detached *N. benthamiana* leaves, placed on moist tissue paper and incubated in sealed boxes. The lesion areas (cm^2^) were measured at 36 h under UV light after inoculation by mycelium plug. The primers (Supplementary Table 2) specific for *P. capsici* and *N. benthamiana* actin gene were used to quantify the relative biomass of pathogen by quantitative PCR. The experiments were conducted in triplicates and were repeated at least three times with a minimum of 18 infected leaves.

### Agro-Infiltration Assay

The pBINGFP2-PsAvr3c, pBINGFP2-PsAvh27b, pBINGFP2-ProbiAvh89, pBINGFP2-PparvAvh214 and RFP-GmSKRP1, pBINGFP2-GmSKRP1 fusion vectors were transformed into *A. tumefaciens* strain GV3101 and then grown overnight at 28°C in Luria-Bertani culture medium with kanamycin and rifampicin antibiotics. The cells were harvested by centrifugation at 5000 rcf and resuspended in *Agro-infiltration* buffer (10 mM 2-[N-morpholino] ethanesulfonic acid [MES], 10 mM MgCl_2_ and 150 mM acetosyringone). The OD600 was adjusted to 0.05–0.5, depending on the experiments, before syringe infiltration into the leaves of 3 to 4-week-old *N. benthamiana* plants. For co-expression of multiple constructs, suspensions carrying each construct were thoroughly mixed before infiltration. The agrobacterial suspension was left at room temperature for 2 h before infiltration.

### Confocal Microscopy

After 2 dpi, patches of *agro-infiltrated* *N. benthamiana* leaves were cut and mounted in distilled water and analyzed using an LSM 710 laser scanning microscope (Carl Zeiss, Germany) with a 20×, 40×, or 60× objective lens. The green and red fluorescence were observed at excitations of 488 nm or 561 nm, respectively.

## Results

### Sequence Analyses of PsAvr3c Effector Family Proteins in *Phytophthora* Species

**FIGURE 1:**
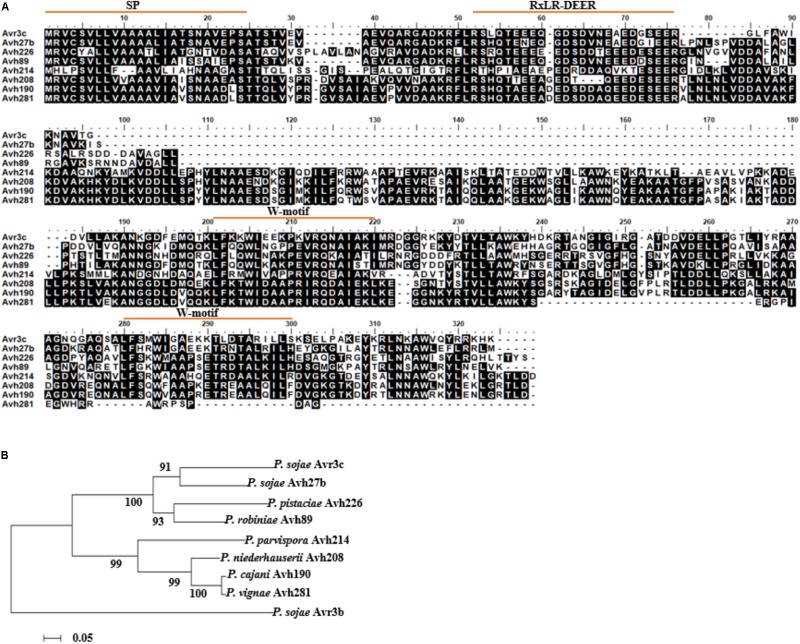
Identification of PsAvr3c family proteins in *Phytophthora* species. **(A)** Full-length sequence alignment of PsAvr3c family proteins from *P. sojae*, *P. cinnamomi* var. *robiniae*, *P. pistaciae*, *P. parvispora*, *P. niederhauserii*, *P. cijani*, and *P. vignae*. The amino acid sequences (Supplementary Table 1) are from NCBI database and unpublished *Phytophthora* genome initiative database; alignment was generated by BioEdit. Identical residues are boxed in black. **(B)** Phylogenetic relationships of *PsAvr3c* homologous genes from various *Phytophthora* spp. Neighbor-Joining analysis of Kimura’s distances calculated based on the nucleotide sequences with the MEGA5 program, using full-length amino acid sequences, numbers indicate bootstrap value from 1,000 replicates. The sequence of *PsAvr3b* was used as an out-group.

The previous study demonstrated that PsAvr3c is a virulence effector which can reprogram host pre-mRNA splicing to promote disease (Huang et al., 2017). Recently we found that potential homologous proteins of PsAvr3c exist in several *Phytophthora* species. To further validate the presence of *PsAvr3c* gene family and make functional analysis, we conducted a BLAST search of *PsAvr3c* homologs in both NCBI database and unpublished *Phytophthora* genome initiative database. *PsAvr3c* homologs were identified from seven *Phytophthora* spp. (*P. sojae*, *P. cinnamomi* var. *robiniae*, *P. pistaciae*, *P. parvispora*, *P. niederhauserii*, *P. cijani*, and *P. vignae*) with an E-value cut-off at 1e-15 (Supplementary Table 1). Among these sequences, only *PsAvh27b* is a paralog from *P. sojae*, while all the others are regarded as *PsAvr3c* orthologs. Multiple sequence alignment analysis revealed that all of these effectors had a signal peptide and RXLR motif, and also contained two conserved W-motifs within the effector domain. However, no other functional domains or NLSs were apparent in the predicted sequences of these effectors (**Figure 1A**).

To investigate evolutionary relationships among *PsAvr3c* and its family members, we ran sequence alignment and conducted an unrooted phylogenetic tree using the neighbor-joining (NJ) algorithm (**Figure 1B**). The results revealed that *PsAvr3c* family members are classed into three main clades, *PsAvr3c* and *PsAvh27b* belong to one clade, *ProbiAvh89* and *P. pistAvh226* form one clade, the others including *P. parvAvh214*, *P. niedAvh208*, *P. cijaAvh190*, and *P. vignAvh281* form another clade. In addition, we also concluded that both *PsAvh27b* and *ProbiAvh89* are the closest genes from PsAvr3c in the phylogenetic tree, and may share the most similar biological functions. *PparvAvh214* together with other homologous are quite distant from *PsAvr3c*, indicating potentially diversified functions from PsAvr3c. On this basis, we focused our research on PsAvh27b, ProbiAvh89, and PparvAvh214, with the aim to support the hypothesis that the effector family members that were most closely related to PsAvr3c from the phylogenetic tree may share the fundamental virulence function, and the gene which was farther related to PsAvr3c differentiated into other virulence functions during evolution.

### PsAvr3c Family Proteins Bind to GmSKRPs *in Vitro* and Promote GmSKRPs Stability in *Planta*

**FIGURE 2:**
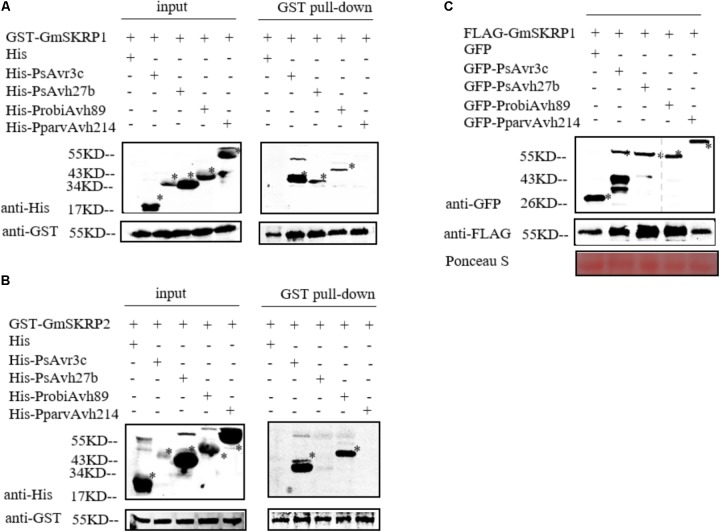
PsAvr3c family proteins bind to SKRP and promote SKRP stability *in planta.* **(A)** PsAvr3c family proteins physically interact with GmSKRP1 *in vitro*. GST-GmSKRP1 or GST bound resins were incubated with *E. coli* supernatant containing His-PsAvr3c, His-PsAvh27b, His-ProbiAvh89, and His-PparvAvh214. Proteins bands of GST-GmSKRP1 are marked by asterisks. The presence of His-tagged proteins was detected by western blot using anti-His tag antibody. **(B)** PsAvh27b and PparvAvh214 don’t bind to GmSKRP2 *in vitro*. GmSKRP2 (*Glyma.19G231600*) shared 94% similarity with GmSKRP1 at the amino acid sequence level. Western blotting showing that His-ProbiAvh89 protein was detected in GST-GmSRKP2 pull-down products, but could not detect clear signals of His-PsAvh27b and His-PparvAvh214 in the presence of GST-GmSKRP2. Protein bands of GST-GmSKRP2 are marked by asterisks. The presence of His-tagged proteins were detected by western blot using anti-His tag antibody. **(C)** PsAvr3c stabilizes GmSKRP1 proteins *in planta*. The FLAG-GmSKRP1 were co-expressed with GFP-PsAvr3c, GFP-PsAvh27b, GFP-ProbiAvh89, GFP-PparvAvh214 or GFP. Total proteins were extracted at 48 h post inoculation (hpi). Immuno-blotting showed an increased signal from FLAG-GmSKRP1 in the presence of GFP-PsAvh27b and GFP-ProbiAvh89 but not with protein GFP-PparvAvh214 or GFP (left lanes). The position of expected protein bands are indicated by asterisks, and protein loading was determined by Ponceau stain.

PsAvr3c physically binds to and stabilize GmSKRPs, a negative immune regulator rich in residues of serine, lysine, and arginine (Huang et al., 2017). GmSKRP1 and GmSKRP2 differ in DNA sequences length, coding 558 bp and 552 bp respectively, but still share 94% similarity at the amino acid sequence level (Huang et al., 2017). To test whether PsAvr3c homologous proteins interact with GmSKRPs, we expressed recombinant His-PsAvh27b, His-ProbiAvh89, His-PparvAvh214, and His-PsAvr3c (as positive control) proteins together with GST-GmSRKP1/2 or GST (as negative control) proteins in *Escherichia coli*, and performed GST pull-down assays. The results (**Figure 2A**) show that both PsAvh27b and ProbiAvh89 were detected in GmSRKP1 pull-down products *in vitro*; however, no band could be detected between PparvAvh214 and GmSRKP1. Surprisingly, ProbiAvh89 was the only one that comes down with GmSKRP2. Neither PsAvh27b nor PparvAvh214 could be detected in the GST-GmSKRP2 pull-down assay (**Figure 2B**). This observation suggests that ProbiAvh89 directly binds to both GmSKRPs as PsAvr3c, while PsAvh27b only binds to GmSKRP1 but not GmSKRP2. However, no direct interaction between PparvAvh214 and GmSKRPs were observed in our assay.

To test whether selected PsAvr3c family effectors stabilize GmSRKP1 *in vivo*, we expressed FLAG-GmSRKP1 together with individual GFP tagged PsAvr3c family members in *N. benthamiana* and harvested protein samples for Co-immunoprecipitation (Co-IP) precisely as we had done in our earlier study (Huang et al., 2017). Immuno-blot data indicated that significant amounts of GmSRKP1 were accumulated in the presence of PsAvh27b or ProbiAvh89 compared to PparvAvh214 and GFP (**Figure 2C**). These results together with our *in vitro* pull-down data suggested that PsAvr3c family proteins interact with GmSKRPs with different binding activities, and provides excellent natural variants to study SKRP mediated plant susceptibility.

### The Alternatively Splicing Pattern of Several Soybean Pre-mRNA Are Changed in the Presence of PsAvr3c Family Effectors

To further investigate whether soybean pre-mRNAs are alternatively spliced by other family members, we expressed PsAvr3c family effectors in individual-soybean hairy roots, and five soybean hairy roots were gathered to extract RNA for measuring the splicing ratio of selected marker genes by qRT-PCR. The marker genes we selected for this study were a NAC transcription factor (*Glyma.02G222300*), a WRKY transcription factor (*Glyma.03G220800*), a histidine-containing phosphotransfer factor (*Glyma.02G150800*) and a control gene called COP9 signalosome complex subunit (*Glyma.03G016800*), which has the intron splicing that is not affected by PsAvr3c and GmSKRP1 as we previously examined in our earlier study (Huang et al., 2017).

**FIGURE 3:**
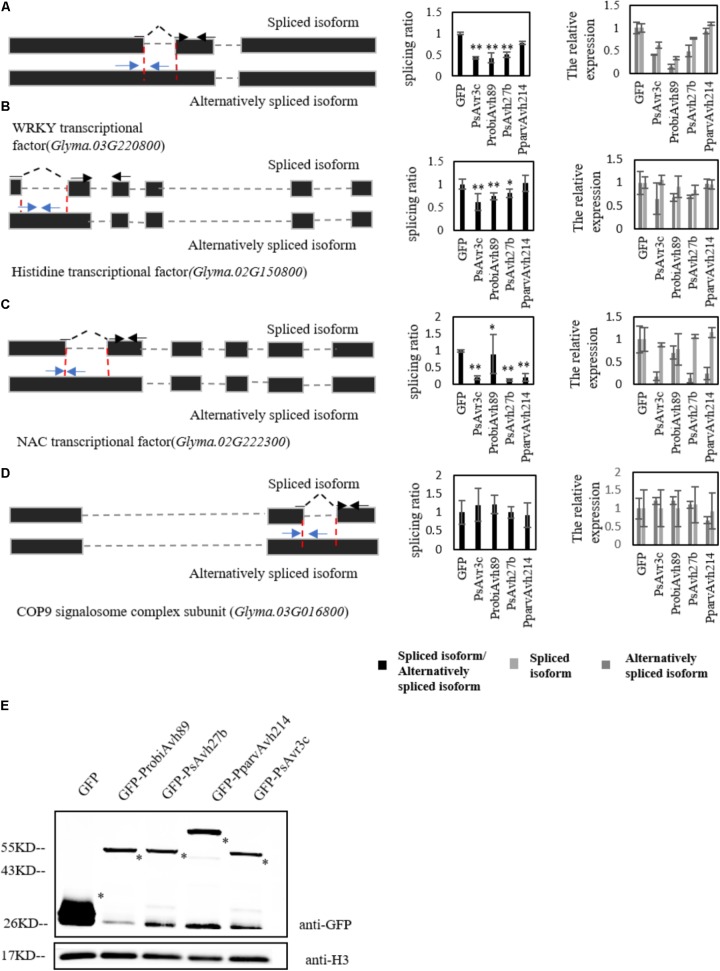
Soybean pre-mRNAs are alternatively spliced by PsAvr3c family effectors. **(A–D)** Analysis by qRT-PCR was performed using specific primers to measure different isoform transcript level of WRKY transcription factor **(A)**, histidine-containing phosphotransfer factor **(B)**, NAC transcription factor **(C)**, and control gene COP9 signalosome complex subunit **(D)**. The left panel shows the schematic gene model; the blue arrows indicate the primers designed to span the intron–exon junction that were used to measure the alternatively spliced isoform. The spliced primers crossing exon–exon junctions are shown with black arrows over the intron. Splicing efficiency ratio was calculated by determining the level of spliced RNA normalized to the level of alternatively spliced RNA. The soybean actin gene CYP2 was used as an internal control gene. Experiments were repeated three times with similar results. Means and standard errors from three replicates are shown (^∗^*P* < 0.05; ^∗∗^*P* < 0.01; one-way ANOVA). **(E)** Immuno-blot analyses of the GFP-PsAvr3c family effectors or GFP proteins in fluorescent hairy roots of soybean cv. Williams. Transformed hairy roots were selected based on the green fluorescence. The total proteins were extracted from the hairy roots for analyses. Recombinant protein expression was confirmed by immuno-blotting using anti-GFP antibody. Asterisks indicate protein bands.

Significant changes in splicing ratio of splicing variants from WRKY transcription factor and histidine-containing phosphotransfer factor were detected by qRT-PCR in ProbiAvh89 and PsAvh27b expressed hairy roots. However, no change was detectable in PparvAvh214 over-expressed lines (**Figures 3A,B**). Unexpectedly, the splicing ratio of the NAC transcription factor gene was significantly altered in all the effector expressing hairy roots (**Figure 3C**). As a control, intron splicing of COP9 signalosome complex subunit gene was not affected in all the lines (**Figure 3D**). Meanwhile, the leftover of the same samples was also used to extract proteins for effector gene expression testing. Immuno-blot data demonstrated that these effector proteins accumulated at a similar level in soybean hairy roots (**Figure 3E**), suggesting that the changes of splicing ratio are not likely due to the gene expression difference. These results demonstrate that ectopic expression of either ProbiAvh89 or PsAvh27b in soybean hairy roots affects the alternative splicing of selected genes in a PsAvr3c-similar manner.

### PsAvr3c Family Effector Enhanced SKRP Mediated Plant Susceptibility With Different Activities

**FIGURE 4:**
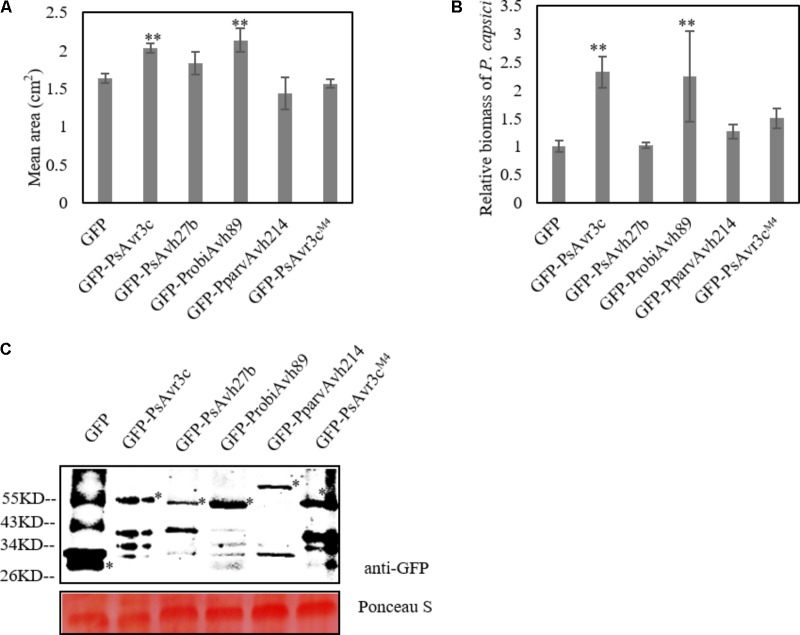
Co-expression of GFP-PsAvr3c family proteins and GFP-PsAvr3c^M4^ with GFP-GmSKRP1 in *N. benthamiana*. **(A)** Co-expression of GFP-PsAvr3c family effectors and GFP-GmSKRP1 in *N. benthamiana* leaves, GFP-PsAvr3C^M4^ mutant was used as the negative control which can not enhance the susceptibility of GmSKRP1. *P. capsici* mycelia were inoculated on the infiltrated leaves at 36 h after *Agro*-infiltration. Lesion areas (cm^2^) were measured at 36 hpi. Means and standard errors from three replicates are shown. Letters represent significant differences as measured (^∗∗^*P* < 0.01; one-way ANOVA). The experiment was performed in triplicates and repeated three times with similar results. **(B)** Relative biomass of inoculated *N. benthamiana* leaves. DNA from *P. capsici* infected leaves was isolated at 36 hpi, and primers specific for the *N. benthamiana* and *P. capsici* actin gene were used for qRT-PCR biomass assay. qRT-PCR was performed and normalized. Means and standard errors from three replicates obtained from three independent experiments that gave similar results are shown (^∗^*P* < 0.05; one-way ANOVA). **(C)** Immunoblot analyses of GFP, GFP-PsAvr3c, GFP-PsAvh27b, GFP-ProbiAvh89, and GFP-PparvAvh214 proteins, and GFP-PsAvr3C^M4^. Total proteins were extracted at 36 hpi. Protein expression was confirmed by immunoblotting using an anti-GFP antibody. Protein bands are indicated by asterisks while protein loading is indicated by Ponceau stain.

Ectopic expression of GmSKRP1 in *N. benthamiana* promotes *Phytophthora capsici* infection, and co-expression of PsAvr3c has a synergistic effect on GmSKRP1 mediated susceptibility (Huang et al., 2017). To investigate whether other family members enhanced GmSKRP1 mediated susceptibility, we co-expressed the individual effector together with GmSKRP1 in plant cells prior to pathogen challenge. As shown in data, only co-expression of ProbiAvh89 with GmSKRP1 resulted in increased plant susceptibility to *P. capsici* (Supplementary Figure 1). However, this synergistic effect exhibited no clear differences when the PsAvh27b and PparvAvh214 were co-expressed with GmSKRP1 respectively (**Figures 4A,B**). Here, PsAvr3c and its GmSKRP binding deficient mutant PsAvr3c^M4^ were used as a positive and negative control, respectively. Proteins of the expected size were detected (**Figure 4C**). Our data indicated that only effector ProbiAvh89 functions in a similar manner to PsAvr3c and promoted plant susceptibility in a GmSKRP1-dependent manner.

### Translocation of GmSKRP From the Nucleoplasm to Nucleolus Is Not Associated With Effector-Mediated Susceptibility and Pre-mRNA Alternative Splicing

**FIGURE 5:**
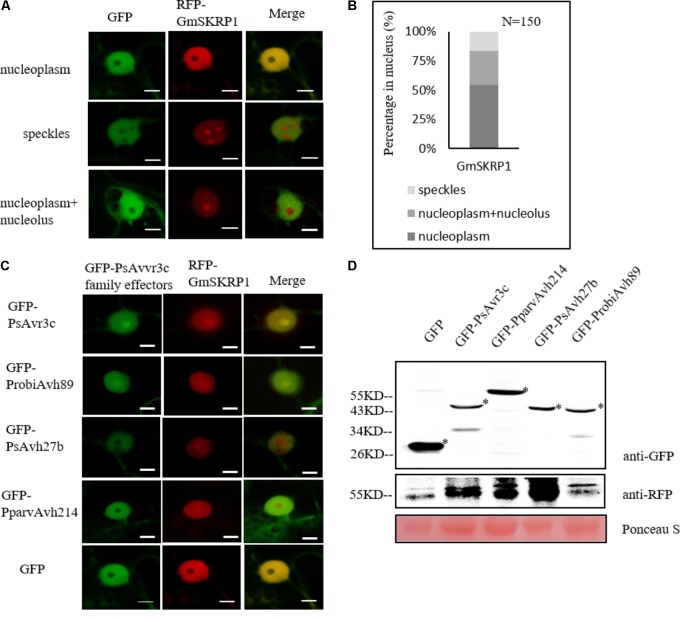
GmSKRP1 are relocated from the nucleoplasm to nucleolus with different PsAvr3c family effectors. **(A)** The localization pattern of RFP-GmSKRP1. Confocal imaging shows that RFP-GmSKRP1 proteins are diffusely distributed in the speckles, nucleoplasm, and mixed pattern including nucleoplasm and nucleolus. Scale bar represents 10 μm. **(B)** GmSKRP1 proteins predominantly (>50%) localized in the nucleoplasm of a total of 150 randomly selected and analyzed cells from *N. benthamiana* plant leaves, 16% proteins accumulated in speckles. **(C)** GmSKRP1 was relocated from nucleoplasm to nucleolus in the presence of PsAvr3c family effectors. Confocal images of *N. benthamiana* leaf epidermal cell nuclei transiently expressing the RFP-GmSKRP1 with GFP-PsAvr3c homologous proteins demonstrate that GmSKRP1 protein were relocated from the nucleoplasm to the nucleolus when they are co-expressed with GFP-PsAvr3c family proteins but not with GFP control. Scale bar represents 10 μm. **(D)** Immunoblot analyses of GFP, GFP-PsAvr3c, GFP-PsAvh27b, GFP-ProbiAvh89, and GFP-PparvAvh214 proteins. Total proteins were extracted at 48 hpi. Protein expression was confirmed by immunoblotting using an anti-GFP antibody. Protein bands are indicated by asterisks while protein loading is indicated by Ponceau stain.

To further investigate the relationship of effector genes with their subcellular localization, we generated the RFP-GmSKRP1 fusion, and GFP tagged effector fusion constructs and introduced them into *N. benthamiana* for confocal observations. When we co-expressed GmSKRP1 with GFP in *N. benthamiana*, GmSKRP1 was diffusely distributed in the nucleoplasm, nuclear speckles and a mixed pattern including nucleoplasm and nucleolus (**Figure 5A**). GmSKRP1 proteins predominantly (>50%) localized in the nucleoplasm of a total of 150 randomly selected and analyzed cells from *N. benthamiana* plant leaves. In other cases, GmSKRP localized in either nuclear speckles or in non-specific pattern (**Figure 5B**). We then co-expressed other family members with GmSKRP1 and checked their subcellular localization. We observed that ProbiAvh89 localized in the nucleolus like PsAvr3c, but both PsAvh27b and PparvAvh214 were excluded from the nucleolus and mainly localized in the nucleoplasm (**Figure 5C**). Interestingly, all of these effectors resulted in the accumulation of RFP-GmSKRP1 protein from the nucleoplasm to the nucleolus (Supplementary Figure 2), a phenomenon that was not observed in GFP control tests (**Figure 5C**). Expression of these fusion proteins were verified by western blot (**Figure 5D**). These findings combined with our previous data, indicate that translocation of SKRP from the nucleoplasm to the nucleolus is not associated with virulence and splicing.

## Discussion

Alternative splicing has been found to be an important regulatory process in global gene expression (Yang et al., 2014). Previously, we reported that *Phytophthora sojae* core effector, PsAvr3c, targets and stabilizes GmSKRP proteins. GmSKRPs are soybean RNA spliceosome-associated proteins that participate in pre-mRNA splicing process, that affect hundreds of soybean pre-mRNA splicing (Huang et al., 2017). SKRP homologous proteins exist in soybean, tomato, *Arabidopsis*, *N. benthamiana*, *Zea mays*, *Oryza sativa* (Huang et al., 2017). However, whether effector-SKRP associations are prevalent in other *Phytophthora* species, and manipulate host plant RNA splicing machinery remains unknown. In the present study, we made the sequences alignment of PsAvr3c homologous, and found that although PsAvr3c and its homologous effector are not highly conserved, important functional motifs are apparent, including the signal peptide, the RxLR and EER motifs in the host-targeting region, and core residues in the two W domains. Although our analysis included just seven PsAvr3c homologous from other *Phytophthora* species, other effectors may also play roles in the manipulation of the pre-mRNA splicing machinery. For instance in *P. capsici*, an Oomycete, with a broad host range, and a robust model for investigations (Lamour et al., 2012). Considering that NbSKRP amino acid sequence shares high similarity with the GmSKRPs, silencing the NbSKRP gene in *N. benthamiana* resulted in significant resistance to *P. capsici* than the control plants, suggesting that SKRP in *N. benthamiana* and soybean confers susceptibility to other *Phytophthora* (Huang et al., 2017). SKRP-effector interactions seem to occur in other *Phytophthora* pathosystems. Therefore, we hypothesized that the pathogen effector interfering with host RNA splicing machinery to reprogram the splicing of plant pre-mRNA is a general pathogenic mechanism. Further experiments are required to validate this possibility.

In our study, we identified both ProbiAvh89 and PsAvh27b that target and stabilize GmSKRP1. In line with these data, our experiments also demonstrate that splicing ratio of WRKY, NAC transcriptional factor and histidine-containing phosphotransfer factor were lower in both ProbiAvh89 and PsAvh27b expression line than control. The ability of the conserved homologous effector to share similar function is not surprising, given that homologous oomycete effectors that employ similar functions have been previously reported. For instance, *Hpa* RXLR effector HaRxL96 and its homologous effector from *P. sojae* PsAvh163 share conserved functional domains and target a central component of the resistance network that contributes to plant resistance, suggesting that conserved effectors manipulate the same or similar targets in the pathogens’ respective hosts (Anderson et al., 2012). Previous studies have demonstrated that expression of *Phytophthora* *sojae* suppressor of RNA silencing (PSR2) significantly enhanced the susceptibility of soybean to *P. sojae*. The homolog of *P. sojae* PSR2 (PsPSR2) in *P. infestans* (PiPSR2), is capable of suppressing RNA silencing activity when expressed in *N. benthamiana* and enhance the susceptibility of *N. benthamiana* to *Phytophthora* infection (Xiong et al., 2014). These discoveries together with our current findings present a compelling case that this mechanism that effector target SKRP protein to interfere with host RNA metabolism is not an isolated phenomenon in *Phytophthora*.

Interestingly, our assay does not detect clear enhancement of the susceptibility when PsAvh27b was co-expressed with GmSKRP1 in *N. benthamiana*, suggesting that the roles of PsAvh27b and PsAvr3c may share similarity but are not entirely redundant. Several pieces of evidence support this hypothesis. Although PsAvh27b target GmSKRP1 in a PsAvr3c similar pattern, there was no apparent binding signal when PsAvh27b was co-incubated with GmSKRP2 in *E. coli*, suggesting that PsAvh27b only binds to GmSKRP1 whereas PsAvr3c and PsAvh89 bind to both GmSKRP1 and GmSKRP2. Hence, the results indicate that both GmSKRP proteins must be affected to obtain a phenotypic effect on disease. Moreover, we noticed that the subcellular localization of PsAvh27b and PsAvr3c are not precisely the same. PsAvh27b predominantly localized at nucleoplasm, while PsAvr3c mainly accumulated in the nucleolus. Therefore, it is possible that although PsAvh27b maintains some similarity with PsAvr3c, it possesses diverse differentiated function. Taken together, that may explain why when only *PsAvr3c* gene was knocked-out in *P. sojae* strain P6497, the virulence of the mutants declined significantly in susceptible soybeans (Huang et al., 2017). The conclusion fits and complements existing theories of neofunctionalization driven by positive selection. Previous studies supports this hypothesis. For example, *Phytophthora sojae* crinkling and necrosis (CRN) gene PsCRN172–2 triggers programmed cell death (PCD) whereas its duplicated copy PsCRN172–1, which only differs by seven amino acids does not (Shen et al., 2013).

In contrast to PsAvh27b and ProbiAvh89, PparvAvh214 showed the opposite effect on the splicing pattern in WRKY transcriptional and histidine-containing phosphotransfer factor pre-mRNA (**Figure 3**), even though it shares 37% identity to PsAvr3c. This suggests that PsAvr3c family effector PparvAvh214 may have differentiated during *Phytophthora* evolution. Indeed, the effector gene family members differentiation into distinct functions have been previously reported. For example, a previous study demonstrated that the evolution of bacterial type III secreted effectors (T3SEs) is strongly influenced by a non-homologous recombination process, that generates novel T3SEs with new virulence functions (Stavrinides et al., 2006). Recent studies have revealed that *Xanthomonas campestris* (Xcv) effector XopD, has a longer N-terminal domain that determines functional specificity in tomato. While XopD family effectors share EAR motifs and SUMO protease activity, they play a specific role within the bacterial-host interaction, and do not complement Xcv ΔxopD mutant phenotypes (Kim et al., 2011).

Interestingly we also found alterations in soybean NAC splicing patterns in the presence of PparvAvh214. One of the possibilities is that PparvAvh214 influences the host plant pre-mRNA splicing through other pathways in a SKRP independent manner. It is still possible that although PparvAvh214 cannot interact with GmSKRPs, it associates with other alternative splicing factors such as SR proteins, thus influencing the splicing ratio of NAC transcription factor. Meanwhile, we did not observe whether PparvAvh214 enhance GmSKRPs induced susceptibility. Another possibility is that SKPRs have natural variations in different plants, thus it is possible that SKRP in the host of *P. parvispora*, such as *A. unedo* (Scanu et al., 2013), is quite different from soybean SKRP. The interaction of PparvAvh214 with its natural host will be important for an in-depth physiological dissection and analysis of its function. Nevertheless, the functional analysis of PsAvr3c family effector illustrates that the mechanism of effector association with SKRP-like proteins is prevalent in plants and provides probes to dissect plant immunity.

## Author Contributions

YZ and JH analyzed the data and contributed constructs. YZ, JH, and SD designed the experiments. YZ, SD, and SO wrote the manuscript. All authors commented on the article before submission.

## Conflict of Interest Statement

The authors declare that the research was conducted in the absence of any commercial or financial relationships that could be construed as a potential conflict of interest.
