# Supplementary material for: Functional Analysis of PsAvr3c Effector Family From Phytophthora Provides Probes to Dissect SKRP Mediated Plant Susceptibility
Source: Front Plant Sci. 2018 Jul 25;9:1105. doi: 10.3389/fpls.2018.01105 (PMC6069499; doi:10.3389/fpls.2018.01105)
Supplement: Supplementary file 1 [file Data_Sheet_1.docx]

Supplementary Material

Functional Analysis of PsAvr3c Effector Family from *Phytophthora* Provides Probes to Dissect SKRP Mediated Plant Immunity

Ying Zhang^1^, Jie Huang^1^, Sylvans Ochola^1^, Suomeng Dong^*, 1, 2^

1. Department of Plant Pathology, Nanjing Agricultural University, Nanjing 210095, China.

2. Key Laboratory of Integrated Management of Crop Diseases and Pests (Ministry of Education), 210095, China.

*** Correspondence:** Suomeng Dong, Department of Plant Pathology, Nanjing Agricultural University, Nanjing, Jiangsu 210095, China.
email:[smdong@njau.edu.cn](mailto:smdong@njau.edu.cn)

**Supplementary Table 1****. A list of PsAvr3c homologous proteins sequence.**

| Gene No. | Protein Sequence |
| --- | --- |
| *P. sojae* Avr3c | MRVCSVLLVAAAALIATSNAVEPSATSTVEVAEVQARGADKRFLRSLQTEEEQGDSDVNEAEDGSEERGLFAWIKNAVTGDVLLAKANKGDFEMQTKLFKKWIEEKPKVRQNAIAKIMRDGGRKKYDTVLTAWKYHDKRTANGIGIRGATDDVDELLPGTLIYRAAAGNQGAQSALFSMWIGAEKKTLDTARILLSKSELPAKEYKRLNKAWVQYRRKHK |
| *P. sojae* Avh27b | MRVCSVLLVAAAALIATSNAVEPSATSTVEVAEVQARGADKRFLRSHQTENEQGDSDVNEAEDGIEERLPNLSPVDDALAGLKNAVKISPDDVLVQANNGKIDMQQKLFQQWLNGPPEVRQNAIAKIMRDGGYEKYYTLLKAWEHHAGRTGQGIGFLGATNAVDELLPQAVISAAAAGDKRAQATLFHRWIGAEEKTRNTALRILHEYGKGILAYTRLNNAWLEFLRRLM |
| *P. pistaciae* Avh226 | MRVCYALLVAAATLIATGNTVDASATAQVVSPLAVLANAGVRAVDADKRLLRSRQTEEEEEDSDDTEEEDESEERGLNVGVVDDAFANLRSALRSDDDAVAGLLPTSTLTMANNGNHDMQRQLFLQWLNAKPEVRQKAIATILRNRGDDDFRTLLAAWMHSGERRTRSVGFHGSNYAVDELLPRLLVKKAGAGDPYAQAVLFSKWMAAPSETRDTALKILHESAQGTRGYETLNAAWISYLRQHLTTYS |
| *P. robiniae* Avh89 | MRVCSVLLVAAAALIAISSAIEPSATSTVKVAEVQARGADKRFLRSHQTEEEQGDSDVNEEEDDSEERGINVDDALAILRGAVKSRNNDAVDALLPHTILAKANNGDFDMQTKLFQQWLKAKPEVRQNAISTIMRNGGYDDYKTLLTAWRYNSERTTSGVGFHGSTKAVDKLLPRGLIDKAALGNVQARETLFGKWIAAPSETRDTALKILHDSGMGKPAYTRLNSAWLRYLNELVK |
| *P. parvispora* Avh214 | MHLPSVLLFAAVLIAHNAAGASTTTQLISSGISPEALQTGIGTRFLRTHPIAEAEPEDRDDAQVKTESEERGIDLKLVDDAVSKIKDAAQNKYAMKVDDLLEPHYLNAAESDKGIQDILFRRWAAAPTEVRKAAISKLTATEDDWTVLLKAWKEYKATKLTAEAVLVPKKADEVLPKSMMLKANDGNHDAQAELFRMWIVAPPRVRQEAIAKVRADVTYSTLLTAWRFSGARDKAGLDMLGYSIPTLDDLLQKSLLAKAISGDVKNQNVLFSRWAAAHQETRDAALKILRDVGKGTDEYSALNNAWQKYLKILGKTLDD |
| *P. niederhauserii* Avh208 | MRVCSVLLVVAAAVIAISNAAEASTTQLVSPRDVSAIAKVQVVNAAKRFLRSHQTTEEAGEDTQEEDESEERTLNLNLVDDAVAKFKDVAKHKYDLKVDDLLSPHYLNAAENDKGIKKILFKRWATAPAEVRESAIKQLAATGEKWSGLLAAWNKYEAKAATGFPVSASVANKADDLLPKSLVAKANGGDLDMQEKLFKTWIDAAPRIRQDAIEKLKESGNTYSTVLLAWKYSGSRSKAGIDELGFPLRTLDDLLPKGALRKAMDGDVREQNALFSQWFAAPKETREAALQILFDVGKGTKDYRALNNAWLNYLEKLGRTLD |
| *P. cajani* Avh190 | MRVCSVLLVAAAAVIAVSNAADLSTTQLVYPRGVSAIAEVPVVDAAKRFLRSHQTAEEADEDSDDAQEEDESEERVLNLNLVDDAVAKFKDVAKHKYDLKVDDLLSPYYLNAAESDSGIMKILFQRWSVAPAEVRKTAIQQLAAKGEKWAGLIKAWNQYEAKAATGFPAPAKIAKTADDLLPKTLVAKANGGDLDVQQKLFKTWIDAAPRIRQDAIEKLKEGGNKYRTVLLAWKYSGARYTAGIDELGVPLRTLDDLLPKGALRKAIAGDVREQNALFSQWVAAPRETREAALQILFDVGKGTKDYRTLNNAWRKYLENLGRTLD |
| *P. vignae* Avh281 | MRVCSVLLVAAAAVIAVSNAADLSTTQLVYPRGVSAIAEVPVVDAAKRFLRSHQTAEEADEDSDDAQEEDESEERALNLNLVDDAVAKFKDVAKHKYDLKVDDLLSPYYLNAAESDSGIMKILFQTWSVAPAEVRKTAIQQLAAKGEKWAGLIKAWNQYEAKAATGFPASAKIAKTADDLLPKTLVEKANGGDLDVQQKLFKTWIDAAPRIRQDAIEKLKEGGNKYRTVLLAWKYSERGPIEGWHRRAWRPSPDAG |

**Supplementary Table 2. Primers used in this study.**

| Primer name | Primer sequence (5′ to 3′) |
| --- | --- |
| PBinGFP-F | AAGACCCCAACGAGAAG |
| PBinGFP-R | GAACCCTAATTCCCTTATCTG |
| PBinGFP-PsAvr3c-F | TACAAGGGTACCCCCATGGTTGAGCCTTCCGCCAC |
| PBinGFP-PsAvr3c-R | GGATCCGTCGACCCCTTACTTGTGTTTCCTTC |
| PBinGFP-Avh27b-F | TACAAGGGTACCCCCATGGAGCCTTCCGCCACGT |
| PBinGFP-Avh27b-R | GGATCCGTCGACCCCTTACATGAGTCGCCGTAGGA |
| PBinGFP -P. robiniaeAvh89-F | TACAAGGGTACCCCCATGGAGCCTTCCGCTACGT |
| PBinGFP -P. robiniaeAvh89-R | GGATCCGTCGACCCCTTACTTGACGAGTTCGTTTA |
| PBinGFP-P. parvisporaAvh214-F | TACAAGGGTACCCCCATGTCCACCACGACACAGTT |
| PBinGFP-P. parvisporaAvh214-R | GGATCCGTCGACCCCTTAGTCGTCCAAAGTCTTGC |
| pET32a-F | TAATACGACTCACTATAGGG |
| pET32a-R | GCTAGTTATTGCTCAGCGG |
| pET32a-PsAvr3c-F | CCGGAATTCCTATTCGCCTGGATCAAAAACGC |
| pET32a-PsAvr3c-R | ATAAGAATGCGGCCGCTTACTTGTGTTTCCTTCGG |
| pET32a-PsAvh27b-F | AAGGCCATGGCTGATATGCTCCCCAACCTGTCGCCT |
| pET32a-PsAvh27b-R | GAATTCGGATCCGATTTACATGAGTCGCCGTAGGAA |
| pET32a-P. robiniaeAvh89-F | AAGGCCATGGCTGATATGGAGCCTTCCGCTACGT |
| PET32a-P. robiniaeAvh89-R | GAATTCGGATCCGATTTACTTGACGAGTTCGTTTA |
| pET32a-P. parvisporaAvh214-F | AAGGCCATGGCTGATATGTCCACCACGACACAGTT |
| pET32a-P. parvisporaAvh214-R | GAATTCGGATCCGATTTAGTCGTCCAAAGTCTTGC |
| PGEX4T-2-GmSKRPs-F | TCCCCAGGAATTCCCATGGCGGCCTCTTCTTCCT |
| PGEX4T-2-GmSKRPs-R | CGCTCGAGTCGACCCCTAATCAAAACTAGCATTTAAAAAG |
| pICH86988-F | GGACACGCTCGAGTATAAGAGCTC |
| pICH86988-R | GGATCTGAGCTACACATGCTCAGG |
| pICH86988-GmSKRPs-FLAG-F | TTTGGTCTCAAATGGATTACAAGGATGACGACGATAAGATGGCGGCCTCTTCTTCCTC |
| pICH86988-GmSKRPs-R | TTTGGTCTCCAAGCCTAATCAAAACTAGCATTTAAAAAG |
| GmCYP2-F | CGGGACCAGTGTGCTTCTTCA |
| GmCYP2-R | CCCCTCCACTACAAAGGCTCG |
| NbActin-qRT-F | ACCATCAATGATCGGAATGGAA |
| NbActin-qRT-R | GCTCATCCTATCAGCAATGCC |
| PC35-Actin-qRT-F | GTACTGCAACATCGTGCTGTCC |
| PC35-Actin-qRT-R | TTAGAAGCACTTGCGGTGCACG |
| Glyma.02G222300-splicedF1 | GACTTGCCTGATTTGTCAGT |
| Glyma.02G222300-splicedR1 | GAGCACGACCGGTATAGA |
| Glyma.02G222300-unsplicedF2 | ATGATGTTTGGGTTATTC |
| Glyma.02G222300-unsplicedR2 | CCAATCCATCAACAGCAC |
| Glyma.03G220800-splicedF1 | CAAAGGAACAGTTGAAGGCT |
| Glyma.03G220800-splicedR1 | GGGCTGTTTTTCACAGCTT |
| Glyma.03G220800-unsplicedF2 | TCCGAACTCTTCATCCAT |
| Glyma.03G220800-unsplicedR2 | ATGCCATGAGCCATTCACA |
| Glyma.03G016800-splicedF1 | AGAGTTAAAGAGTAAAGTTA |
| Glyma.03G016800-splicedR1 | CTTGAGTAAAAATCATTTATC |
| Glyma.03G016800-unsplicedF2 | AACAGGACCTGAACTGGGAA |
| Glyma.03G016800-unsplicedR2 | CTGCCAGATATTGAAAAAG |
| Glyma.02G150800-splicedF1 | GTTTCAACAGGGTTTTTTGG |
| Glyma.02G150800-splicedR1 | GATGAGCATGAGCATCTATT |
| Glyma.02G150800-unsplicedF2 | GTTTCAACAGGTTCTCTTGT |
| Glyma.02G150800-unsplicedR2 | ACCTTGCAGTCCAAGAGTCT |

**Supplementary Figure 1**

**
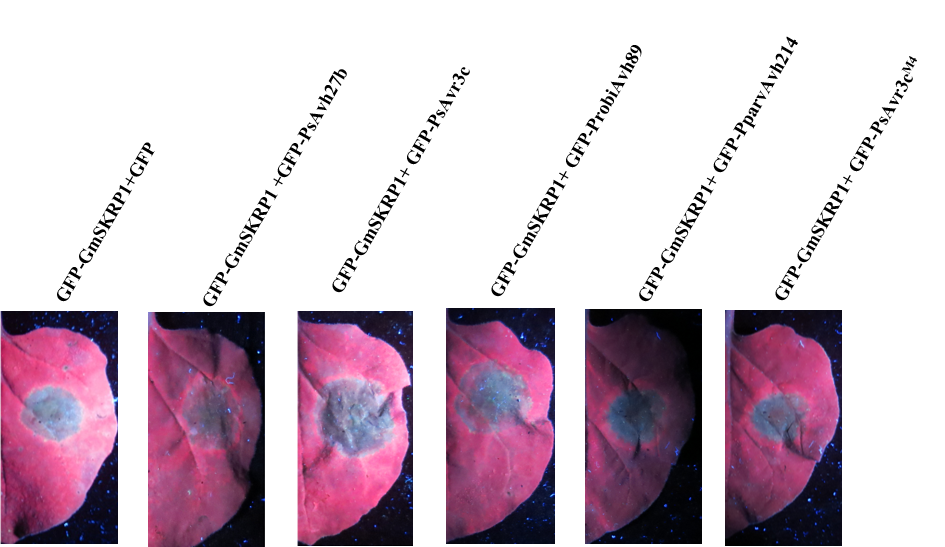
**

**Supplementary Figure 1. Co-expression of GFP-PsAvr3c** **family proteins and** **GFP-PsAvr3c^M4^ with GFP-GmSKRP1 alters *Phytophthora* infection in *N. benthamiana.***

Representative infection lesions from *Phytophthora* inoculated *N. benthamiana* leaves are demonstrated. Agar plot with fresh *P. capsici* mycelia were inoculated on leaves at 36 h after *Agro-infiltration*. Co-expression of GFP-PsAvr3c family proteins and GFP-PsAvr3c^M4^ with GFP-GmSKRP1 in leaves, and ProbiAvh89 results in greater susceptibility to *P. capisici* compared to GFP. Photos were taken at 36 hpi under UV light. Three independent experiments gave similar results.

**Supplementary Figure 2**


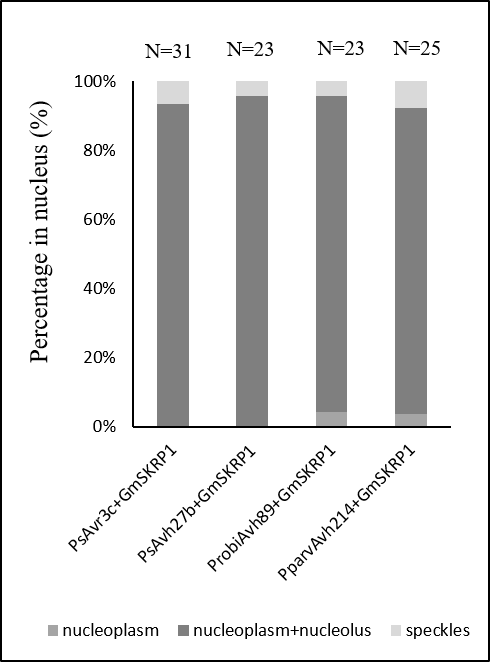


**Supplementary Figure 2.** **GmSKRP1 was relocated from nucleoplasm to nucleolus in the presence of PsAvr3c family effectors**

GFP-PsAvr3c family proteins and RFP-GmSKRP1 are co-expressed in *N. benthamiana*. The statistic analyses of the subcellular localization of RFP-GmSKRP1 fusion protein in the presence of GFP-PsAvr3c family proteins are analyzed.
